# The Relationship Between Obesity Status and Body Image Dissatisfaction on Gross Motor Skill Development and Cardiorespiratory Fitness in Children Aged 6–12 Years Old

**DOI:** 10.3390/ijerph22030417

**Published:** 2025-03-12

**Authors:** Maxime Allisse, Isabelle Thibault, Dominic Gagnon, Emilia Kalinova, Georges Larivière, Mario Leone

**Affiliations:** 1 Faculty of Physical Activity Sciences, University of Sherbrooke, Sherbrooke, QC J1K 2R1, Canada; maxime.allisse@usherbrooke.ca; 2 Department of Health Sciences, University of Québec in Chicoutimi, Saguenay, QC G7H 2B1, Canada; 3 Faculty of Education, University of Sherbrooke, Sherbrooke, QC J1K 2R1, Canada; isabelle.thibault@usherbrooke.ca; 4 Jonquière Médic, Saguenay, QC G7X 7W6, Canada; dominic.gagnon.med@ssss.gouv.qc.ca; 5 Faculty of Sciences, University of Québec in Montréal, Montréal, QC H2X 1Y4, Canada; kalinova.emilia@uqam.ca; 6 School of Kinesiology and Physical Activity Sciences, University of Montréal, Montréal, QC H3T 1J4, Canada; georgeslariviere111@sympatico.ca; 7 Faculty of Medicine and Health Sciences, University of Sherbrooke, Sherbrooke, QC J1H 5N4, Canada

**Keywords:** children, gross motor skills, VO_2_peak, obesity status, body image satisfaction

## Abstract

Background: The harmonious development of gross motor skills (GMSs) is vital for children, fostering their physical, cognitive, and socio-emotional growth. This study aimed to achieve three primary objectives: (1) to establish standardized reference values for all GMS tests conducted; (2) to examine the impact of overweight and obesity on factors influencing the development of GMSs and cardiorespiratory fitness (CRF); and (3) to investigate the relationship between GMSs and CRF levels and body image dissatisfaction among Canadian children from the province of Québec. Methods: The study encompassed 3144 children aged 6 to 12 years (1535 boys and 1609 girls) recruited from 24 elementary schools situated in five urban areas. Anthropometric measurements included body mass, body height, and body mass index (BMI). Physical performance was assessed using a maximal aerobic power test and 12 GMS tests, which comprised two segmental speed tests, four agility tests, two static balance tests, one simple reaction time test, and three coordination tests. Body perception and body image dissatisfaction were evaluated using a silhouette scale featuring two sets of nine drawings depicting a spectrum of body shapes ranging from very thin to obese. Results: Standardized normative values were established for each GMS test. GMSs demonstrated continuous improvement throughout childhood, albeit with a deceleration in progress during later developmental stages. At comparable age, boys generally outperformed girls on tests demanding greater strength, speed, or endurance, whereas girls exhibited superior performance in balance and hand–foot coordination tasks (*p* ≤ 0.05). However, segmental speed remained equivalent between sexes (*p* > 0.05). GMS and CRF were significantly influenced by obesity status. Children with a normal BMI demonstrated superior performance compared to their overweight or obese counterparts, particularly in tests requiring body mass displacement (*p* ≤ 0.05). Conversely, socioeconomic status exhibited no significant impact on body perception in boys (*p* = 0.106), but it was a notable factor among 6–8-year-old girls from lower socioeconomic backgrounds (*p* = 0.045). Conclusions: Obesity status is linked to diminished GMS performance, especially in tasks involving body mass movement. These findings underscore the importance of early intervention strategies to encourage an active lifestyle and promote a healthy body composition in children.

## 1. Introduction

Harmonious acquisition of gross motor skills (GMSs) is essential for optimal child development, particularly before puberty [1,2]. Delays in motor development can have significant consequences, not only in the short term but also in adulthood, limiting participation in various daily activities, including professional work [3,4,5,6,7]. The lack of participation in physical activities is concerning because inactive children are more likely to remain inactive as adults [8], and inactive parents tend to raise their children in similar environments [9,10]. This can lead to an intergenerational cycle of physical inactivity, negatively affecting both physical and mental health. Additionally, early life experiences play a critical role in motor and neurodevelopment as well as the adoption of healthy lifestyles [11]. Children with low motor skills are more likely to adopt a sedentary lifestyle, further limiting their opportunities to enhance their motor abilities [1,12,13]. This creates a vicious circle, where the lack of motor stimulation leads to an increasing disinterest in physical activity, deteriorating both physical condition generally expressed by cardiorespiratory fitness and motor skills [13,14,15]. Although various factors can hinder the typical development of GMSs, the recent epidemic of childhood obesity has become a major concern.

Overweight and obesity, increasingly prevalent in childhood, affect physical and metabolic health and can also hinder motor skills development, which is essential for well-being and an active lifestyle [14,16,17]. This makes childhood obesity a significant concern. While this trend is observed worldwide, a recent study in Québec (Canada) found that more than 16% of children aged 6 to 12 are overweight or obese—more than three times the rate of the 1980s [18]. Several studies have linked obesity to delays in GMS development [1,12,13,14,16]. However, the evaluation of motor skills in children is challenging due to the wide variety of available measurement tools and the lack of consensus on standardized reference criteria. The preference for specific assessment tools often varies depending on geographic region, country, ethnicity, and the socioeconomic status of participants. The use of diverse assessment methods across different studies and countries makes direct global comparisons challenging. Moreover, there is no universal agreement on what could be considered a “gold standard” for assessing motor skills. Additionally, the motor skills expected of children are heavily influenced by sociocultural factors, resulting in significant variations worldwide [19]. This underscores the importance of considering the specific characteristics of the target population. In Canada, the lack of large-scale comprehensive data makes it difficult to determine whether this trend is also prevalent here. Additionally, the absence of standardized, representative normative values limits the effective monitoring of GMSs in Canadian children.

Despite this great heterogeneity, certain similarities seem to emerge from the scientific literature. Indeed, the impact of obesity is particularly evident in tasks requiring rapid or prolonged body movements, where obese children underperform compared to healthy-weight peers. In contrast, motor skills that do not involve significant body mass displacement seem to be less affected [20,21,22]. Excess weight especially affects balance, even without major movements. Overweight or obese children often perform poorly on balance tests due to morphological changes, such as uneven weight distribution, collapsed arches, and increased foot instability. These alterations in postural control elevate the risk of falls and restrict participation in physical activities [20,21,22]. Rodrigues, in 2016, also suggests a bidirectional relationship, indicating that early motor difficulties are not only a result of obesity but can also contribute to its development [23]. The development of motor skills in children is influenced by both biological factors (such as genetics, gender, and maturation) and environmental factors (e.g., educational style, stereotypes, experiences, play opportunities, encouragement, demographics, and social factors) [12,24,25], as well as interactions between these factors [26]. The impact of obesity on motor skills also seems to vary depending on age and the extent of excess weight. Certain studies highlight significant differences in motor performance between overweight and obese children, with overweight children generally being less impacted than their obese counterparts [15]. When examining the relationships between GMSs, physical activity, and physical fitness, weight was found to be strongly associated with age and gender in tests of GMSs [27,28]. Indeed, disparities in gross motor coordination appear more pronounced in older children (ages 10–12) compared to younger children (ages 5–7), suggesting that these limitations become progressively more pronounced over time [29,30].

Beyond the physical aspect, motor competence is also influenced by psychosociological factors. In this context, an area that remains underexplored relates to body image satisfaction and its influence on the development of motor skills. Body image is a multidimensional concept encompassing the cognitions, emotions, attitudes, and behaviors an individual holds toward their own body [31,32,33,34]. It exists on a continuum, ranging from a positive body image to body dissatisfaction [32,33]. A positive body image is characterized by appreciation, acceptance, and respect for one’s physical appearance [32,33,34,35,36,37,38]. Conversely, body dissatisfaction refers to a pronounced dissatisfaction with one’s body shape, accompanied by persistent and pervasive negative thoughts, emotions, and attitudes [32,39]. It arises from a discrepancy between one’s perceived body image and the ideal body image, which is shaped by the internalization of societal norms regarding physical appearance (e.g., thinness) [40,41,42,43,44,45,46,47]. Body dissatisfaction carries significant social costs [48] and has profound implications for well-being, including low self-esteem, academic difficulties, depression, anxiety, eating disorders, impaired social and family functioning, and obesity [33,44,45,49,50,51,52,53,54]. Alarmingly, this issue affects children of all genders without distinction [55,56,57,58,59,60]. Previous studies have established a link between motor skills and obesity in children. Specifically, overweight and obese children exhibit lower fine and GMSs compared to their age-matched peers with a healthy weight. Moreover, these disparities tend to widen with age [15,16,61,62,63,64].

Obesity and overweight are undeniably associated with body dissatisfaction, as highlighted by numerous studies [65,66,67,68,69]. In fact, body dissatisfaction is considered one of the most significant psychological consequences of obesity [70]. Given the well-established links between obesity and motor skills, as well as between obesity and body dissatisfaction, it is crucial to explore the relationship between motor skills and body dissatisfaction. Unfortunately, the existing scientific literature provides limited insights into this issue. The few studies available indicate that both motor skills and body dissatisfaction are consequences of obesity. Indeed, obese children typically exhibit less developed motor skills and higher levels of body dissatisfaction [71,72]. This trend is particularly evident among obese boys, who demonstrate greater body dissatisfaction and lower motor skills compared to their normal-weight counterparts [73]. A study conducted in 2002 revealed that these findings are not exclusive to obese or overweight children. Negative body image and poor self-esteem affect physical activity in a similar way as a well-known variable in the field, which is the poor perception of motor skills [74,75,76,77,78].

Thus, this study pursued three main objectives: (1) to update the standard reference values for all GMS assessment tests used; (2) to examine the impact of overweight and obesity on the factors influencing the development of GMSs in Canadian children from Quebec; and (3) to determine the relationship between the level of motor competence and dissatisfaction with body image.

## 2. Materials and Methods

### 2.1. Design

This cross-sectional epidemiological study examined a large sample of children aged 6 to 12 years old. Data come from a larger study conducted by our research team through a regional, school-based survey conducted between 2014 and 2017.

#### Participants

The sample included 3144 French-speaking students (1535 boys and 1609 girls) with a mean age of 9.5 ±1.8 years enrolled in 24 elementary schools (grades 1 to 6) from 5 urban regions of Québec (Canada). All assessments (anthropometric, GMS, and body image perception) were conducted indoors between 9:00 AM and 3:00 PM, Monday through Friday, from October to May. Measurements were conducted under the supervision of experienced researchers by a team of highly qualified kinesiology interns who had completed 45 h of specialized training. These assessments were carried out during physical education classes, adhering to strict ethical guidelines and following the acquisition of informed consent from parents and students.

A concerted effort was made to ensure the proper representation of various socioeconomic statuses (SESs) within our sample. To achieve this, the Québec Ministry of Education annually develops a deprivation index for each school within its jurisdiction. A school’s socioeconomic status is determined by the proportion of families with children whose income is near or below the low-income threshold. This index is based on a discrete scale ranging from 1 to 10, where 1 represents the most advantaged environments and 10 represents the most disadvantaged ones. Accordingly, schools ranked between 1 and 5 were classified as high-income, while those ranked between 6 and 10 were categorized as low-income. This approach allowed us to capture a diverse range of socioeconomic contexts, ensuring the robustness and inclusivity of our study. The project was approved by the university institutional ethics committee (project number: 602-225-01), and written consent was obtained from school authorities.

### 2.2. Anthropometric Measures

Anthropometric measurements were collected adhering to standardized protocols as outlined by Lohman et al. [79]. Body mass (BM) was determined using a Detecto scale (Webb City, MO, USA) with a precision of 0.1 kg. Body height (BH) was measured using a portable SECA stadiometer model 213 (Hamburg, Germany) with an accuracy of 0.1 cm. Body mass index (BMI) was calculated using the following formula: BM (kg)/BH (m^2^). BMI obesity status classification (typical, overweight, obese) for children was categorized according to age and sex, following the guidelines established by Cole et al. [80].

### 2.3. Cardiorespiratory Fitness Test

Cardiorespiratory fitness (CRF) was assessed using the 20 m shuttle run test, adhering to the protocol outlined and validated by Léger and colleagues [81]. Briefly, the test was conducted in gymnasiums with a minimum length of 25 m. Approximately twenty students typically lined up at the starting line. Commencing at level 1 (corresponding to a running speed of 8.5 km/h), the test progressed with a 0.5 km/h speed increase every minute. Children continued until they were unable to maintain the required pace, at which point the test was stopped, and the last completed stage was recorded. Subsequently, estimated peak oxygen uptake (VO_2_peak) was calculated based on the original formula proposed by the authors in 1988.

### 2.4. Body Image Perception

Children’s body image perception was assessed using a silhouette scale developed by Thompson and Gray [82]. This scale comprises two sets of nine silhouettes depicting a range of body shapes, from very thin to obese, for both male and female figures. First, children selected the silhouette of their own gender that they felt best reflected their current body appearance. Subsequently, they chose the silhouette they would ideally like to embody. The discrepancy between these two selections served as a measure of body dissatisfaction: a negative discrepancy indicated a desire to be thinner, while a positive discrepancy reflected a preference for a more robust physique. In order to verify the validity of the self-assessments of body image, kinesiology trainees conducted independent evaluations of each child. This approach facilitated a comparison between subjective and objective assessments, thereby validating the children’s ability to accurately self-assess their body image.

### 2.5. Gross Motor Skill Tests

Designed for children aged 6 to 12, each test was chosen based on strict criteria. Each of the five selected factors (segmental speed, agility, static balance, coordination, and simple reaction time) had to be assessed using a sufficient number of tests to enable the most comprehensive possible evaluation of the GMS variables. Furthermore, their validity and reliability have been previously demonstrated in research conducted by other scholars [83,84,85]. Reliability was assessed using either test–retest procedures or inter-rater reliability methods, with all correlation coefficients exceeding 0.70, indicating strong consistency.

Validity was established through construct validation, primarily via factor analyses, a widely accepted approach for evaluating children’s motor skills [86]. Furthermore, given the practical constraints of the study, the chosen tests needed to be suitable for application within a school setting. To ensure feasibility for large-scale implementation, several criteria were considered: (1) brevity of administration time; (2) minimal space requirements; (3) ease of understanding for children in the target age group; and (4) affordability and accessibility of necessary materials. To navigate the vast array of available assessment tools, a ten-member expert committee was convened to provide recommendations. Finally, all selected tests were required to yield a quantitative result (a time, a number of points, a frequency of movements, a VO_2_peak value in ml/kg/min, or a number of 1 min stages completed). Based on these rigorous criteria, twelve GMS tests, one cardiorespiratory fitness test, and one functional aerobic capacity assessment were ultimately selected.

#### 2.5.1. Segmental Speed (2 Tests)

(A) One-Hand Tapping

**Figure 1:**
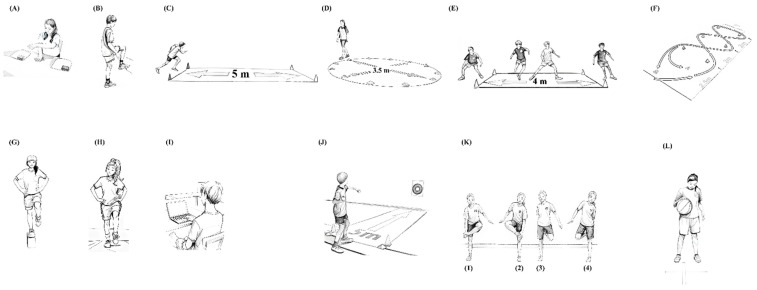
Illustration of the twelve tests used to assess gross motor skill performance in children: (**A**) one-hand tapping; (**B**) two-foot tapping; (**C**) 5 m shuttle run; (**D**) circle run; (**E**) sidestep run; (**F**) slalom run; (**G**) balance eyes opened; (**H**) balance eyes closed; (**I**) simple reaction time; (**J**) target ball toss; (**K**) hand–foot coordination; (**L**) ball dribbling.

This test measures the speed of horizontal arm movements. The child sits at a table with two 20 cm circles spaced 60 cm apart (Figure 1A). The non-dominant hand is stationary between the two circles. The dominant hand touches alternately the center of each circle repeatedly for 20 s. The final score was determined by the number of touches completed within 20 s.

(B) Two-Foot Tapping

This test assesses the ability to execute rapid hip flexion and extension movements. Positioned facing a wall marked with a 30 cm^2^ square, the child must flex one leg to a 90-degree angle and touch the center of the square twice with the tip of the foot (Figure 1B). This sequence is repeated with the opposite leg. The score was determined by the number of “double touches” completed within 20 s.

#### 2.5.2. Agility (4 Tests)

(C) 5 × 5 m Shuttle Run

This test measures the ability to rapidly change direction while running. Two parallel lines, 5 m apart, are marked on the ground. On a signal, the child must sprint 5 m, cross the line with both feet, execute a sharp 180-degree turn, and sprint back to the starting line (Figure 1C). This is repeated five times for a total distance of 25 m. The performance is timed, and the results are recorded with an accuracy of 0.1 s.

(D) Circle Run

This test is designed to assess the ability to quickly and continuously change running direction. A circle with a diameter of 3.5 m is marked on the ground, preferably using cones for clear boundaries (Figure 1D). A starting point is indicated by a line drawn on the ground. The child must complete five full laps around the circle as quickly as possible, choosing the direction of movement. A penalty of 0.5 s is added for each instance where the child touches or crosses the boundary line of the circle. The total time taken to complete the task is recorded with an accuracy of 0.1 s.

(E) Sidestep Run

This test assesses the ability to move laterally as quickly as possible. Two parallel lines are drawn 4 m apart. The child starts with both feet positioned behind one of the two lines. The task is to do sidestep movements from one line to the other, covering a distance of 4 m, repeated 5 consecutive times for a total distance of 20 m (Figure 1E). At each end, the child must touch the line with the nearest foot before changing direction. Leg crossing is prohibited, and the child must maintain a forward-facing position toward the assessor. The time taken to complete the 20 m distance is recorded to the nearest 0.1 s.

(F) Slalom Run

This test assesses agility by measuring the ability to slalom around obstacles as fast as possible. Six cones are arranged as shown in Figure 1F. Two parallel rows of cones should be spaced 2 m apart in width. Along the length of the course, the starting line is positioned 2.5 m from the first cone, with each subsequent cone spaced 2.0 m apart. The child must run as quickly as possible to their right, weaving around the cones in a slalom pattern. After completing the course, without stopping, they must repeat it following trajectory A and then finish the test by crossing the starting line (B). The total time is recorded with an accuracy of 0.1 s.

#### 2.5.3. Balance (2 Tests)

(G) Balance Eyes Opened

The purpose of this test is to evaluate the ability to maintain a unipedal stance with eyes opened on the dominant leg. The child is positioned on a wooden beam measuring 9 cm x 4 cm x 75 cm and instructed to maintain balance as long as possible with their hands on their hips (Figure 1G). The test ends when the child touches the ground, the beam, or the supporting leg with the free leg or if one or both hands are removed from the hips. The total time balanced, up to 60 s, is measured to the nearest 0.1 s.

(H) Balance Eyes Closed

The same procedure must be conducted with eyes closed, this time with the dominant leg standing directly on the ground (Figure 1H). The stopping criteria are the same as those for the eyes-open test, with two additional conditions: the prohibition of opening the eyes and any pivoting of the foot on the supporting leg. The maximum time is set at 60 s, and the result must be recorded with an accuracy of 0.1 s.

#### 2.5.4. Reaction Time (1 Test)

(I) Simple Reaction Time

This test evaluates the ability to react quickly to a visual stimulus. Using a computer program specifically designed for this study, the child had to react as quickly as possible to the appearance of a visual signal (a green triangle displayed on the center of the screen) by pressing the space bar using his dominant hand (Figure 1I). The child is required to complete 25 trials, with reaction times ranging between 100 milliseconds (minimum) and 350 milliseconds (maximum). The result is calculated as the average of the 25 successful trials.

#### 2.5.5. Coordination (3 Tests)

(J) Target Ball Toss

This test evaluates hand–eye coordination through a precision throw. Standing five meters from a 60 cm target (with a 20 cm center) placed 120 cm high, the child throws a tennis ball overhand (Figure 1J). Ten throws were permitted. Each successful hit earns one point, with two points awarded for hits within the target’s center. Crossing the starting line with the feet is not allowed. The final score was the total number of points accumulated, with a maximum of 20 points.

(K) Hand–Foot Coordination

This test is designed to evaluate the ability to move the upper and lower limbs alternately and synchronously as quickly as possible. The test follows the sequence illustrated in Figure 1K: touch the left foot with the right hand while bending the leg forward (1); repeat the same movement with the right foot and left hand (2); touch the right foot with the left hand while bending the leg backward (3); and repeat the same movement with the left foot and right hand (4). This sequence (1 to 4) constitutes one complete cycle. The result is determined by the time taken to complete four consecutive cycles, measured with an accuracy of 0.1 s.

(L) Ball Dribble

This test is designed to evaluate the ability to dribble effectively a ball using their dominant hand. The child stands with their legs slightly bent and shoulder-width apart. The ball must be dribbled continuously in front of the participant and remain in the space defined by their two feet. The objective was to perform as many dribbles as possible within 20 s (Figure 1L). Additionally, with each bounce, the ball must rise to at least hip height.

With the exception of simple reaction time (averaged across 25 trials) and target ball toss (score based on 10 throws), children were given two attempts per test, with only the best result recorded.

#### 2.5.6. Statistical Analysis

Descriptive statistics are presented as means ± standard deviations with 95% confidence intervals (CIs). Group comparisons were conducted using analysis of variance (ANOVA) or independent samples Student’s t-tests. Spearman’s and Kendall’s Tau-b correlation coefficients were employed to assess the agreement between raters’ and children’s evaluations of body image perception. Cohen’s d or *f* effect sizes were calculated to quantify the magnitude of observed group differences. The normality of distributions was determined by means of the Shapiro–Wilk test. In cases of non-normality, a Box–Cox transformation was applied. Growth curves were fitted using cubic splines based on the Box–Cox power exponential method recommended by the WHO [87]. Outliers were identified using the method of Hoaglin and Iglewicz [88], and percentiles were calculated using the Cole and Green LMS method [89]. For a detailed description of the methodology, please refer to our previous publications.

## 3. Results

Table 1 presents the anthropometric, the GMS, and the cardiorespiratory fitness characteristics of boys and girls who participated in this study. No significant differences were found between sexes for age, BM, BH, BMI, or the one-hand and two-foot tapping tests. However, significant sex differences emerged in other variables. Boys outperformed girls in tasks involving BM movements, such as all running tests, including the 20 m shuttle run, as well as in simple reaction time, target ball toss, and ball dribbling. Conversely, girls exhibited superior performance in the two static balance tests and hand–foot coordination. Most effect sizes indicated a small to moderate clinical relevance in explaining these sex differences. For detailed information by year of chronological age for both sexes, refer to the Supplementary Materials.

**Figure 2:**
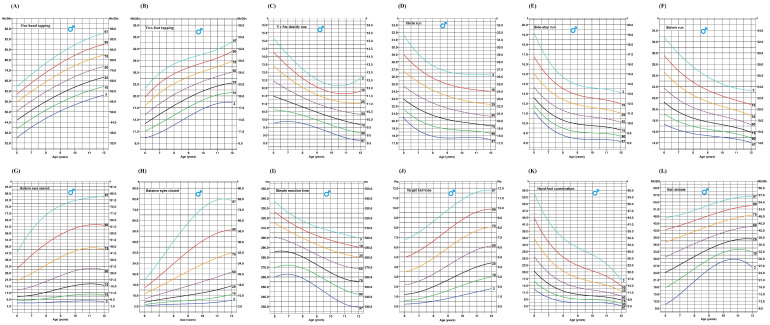
Age-specific smoothed percentile curves for (**A**) one-hand tapping, (**B**) two-foot tapping, (**C**) 5 × 5 m shuttle run, (**D**) circle run, (**E**) sidestep run, (**F**) slalom run, (**G**) balance with eyes opened, (**H**) balance with eyes closed, (**I**) simple reaction time, (**J**) target ball toss, (**K**) hand–foot coordination, and (**L**) ball dribbling for boys aged 6–12 years.

**Figure 3:**
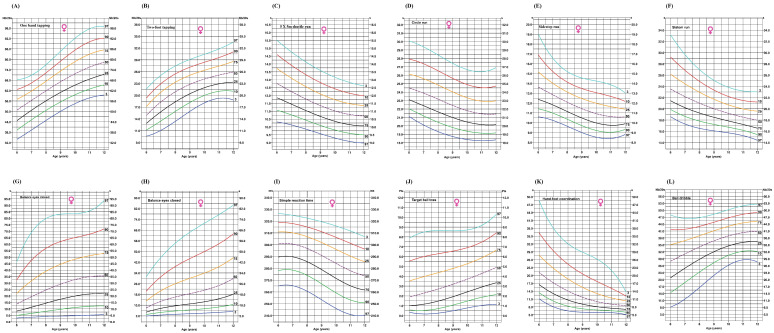
Age-specific smoothed percentile curves for (**A**) one-hand tapping, (**B**) two-foot tapping, (**C**) 5 × 5 m shuttle run, (**D**) circle run, (**E**) sidestep run, (**F**) slalom run, (**G**) balance with eyes opened, (**H**) balance with eyes closed, (**I**) simple reaction time, (**J**) target ball toss, (**K**) hand–foot coordination, and (**L**) ball dribbling for girls aged 6–12 years.

Figure 2 and Figure 3 present smoothed age-specific percentile curves for various motor skill tests, including one-hand tapping, two-foot tapping, the 5 × 5 m shuttle run, circle run, sidestep run, slalom run, balance with eyes open, balance with eyes closed, simple reaction time, target ball toss, hand–foot coordination, and ball dribbling. The purple dotted line indicates the median value for each marker across different ages.

Table 2 compares the GMSs and cardiorespiratory fitness profiles of children of both sexes based on their body image satisfaction. Overall, children with a positive perception of their body image tend to perform better in most tests than their dissatisfied counterparts. Boys dissatisfied with their body image appear to be more significantly affected than girls in the same situation. For instance, in terms of cardiorespiratory fitness, dissatisfied boys exhibit significantly lower VO_2_peak values and functional performance (number of stages completed), a pattern that is not observed among girls.

Table 3 provides a detailed analysis of body dissatisfaction among children, emphasizing its links to factors such as gender, obesity status, and socioeconomic background. The results reveal a generally low satisfaction rate, with only 43.2% of boys and 42.7% of girls reporting satisfaction with their body image. A pronounced gender difference emerges, with a higher percentage of girls (47.1%) desiring thinness compared to boys (31.7%). Among dissatisfied children, 82.3% of girls express a desire to be thinner, whereas this sentiment is shared by only 44.2% of boys. Notably, nearly half of the children with a typical BMI also report dissatisfaction with their body image. As expected, dissatisfaction increases dramatically among obese children, surpassing 81.4% in boys and 84.7% in girls. Socioeconomic status, however, does not significantly influence body image satisfaction in either boys (*p* = 0.106) or girls (*p* = 0.748). Finally, self-assessments of body image prove reliable, demonstrating strong and significant correlations with independent raters (r = 0.558, Kendall’s Tau-b = 0.474 for boys; r = 0.624, Kendall’s Tau-b = 0.542 for girls).

Similarly, Table 4 and Table 5 highlight a notable trend in body image satisfaction across different age groups. Overall, body image satisfaction increases with age, rising from 39.4% to 45.3% between the ages of 6–8 and 9–12 for boys, with a similar pattern observed among girls. Among boys dissatisfied with their body image at 6–8 years old, approximately half expressed a desire for increased stoutness, a proportion that rises to 60% among 9–12-year-olds. Conversely, among girls, the desire for a more robust physique diminishes slightly with age, decreasing from 23.4% to 14.9%. For boys with a typical BMI, 40% of those aged 6–8 report being satisfied with their body image, increasing to 50.8% at the age of 9–12 years.

A similar trend was also observed among girls. Socioeconomic status (SES) appears to exert a differential influence on boys and girls. While it has no discernible impact on boys, a notable disparity is observed among 6–8-year-old girls, which subsequently disappears. In general, older children seem to have a greater capacity for objectively assessing their body image. Nonetheless, the overall results remain highly positive.

Table 6 reveals a significant negative impact of obesity status on both GMSs and cardiorespiratory fitness in children aged 6–12 years. While the magnitude of these effects varied slightly between boys and girls, dynamic tests were consistently affected, particularly VO_2_peak and functional capacity as expressed by the number of stages completed in the 20 m shuttle run test (*f* = 0.53 and 0.61 for boys and *f* = 0.36 and 0.44 for girls). In contrast, static tests showed limited sensitivity to obesity status in both sexes.

Table 7 highlights the impact of body image satisfaction on GMS performance and cardiorespiratory fitness in children with a typical BMI. The findings indicate that most GMS components are significantly influenced by body image perception, with children dissatisfied with their body image demonstrating less favorable outcomes compared to their satisfied peers. In contrast, at comparable BMI levels, body image satisfaction does not appear to significantly affect cardiorespiratory fitness in girls. In boys, however, only aerobic functional capacity, as measured by the number of stages completed during the 20 m shuttle run test, appears to be significantly impacted.

## 4. Discussion

Based on a representative sample of 3144 Canadian children residing in Québec, this study provides innovative insights into various aspects of GMS development, cardiorespiratory fitness, and body image satisfaction within this population. Notably, it facilitated the update of normative values for GMS assessment tests, an endeavor that, to our knowledge, marks a first in Canada with a sample of this magnitude. Moreover, the study underscores the need for further investigation into the complex interplay between these factors, particularly the impact of obesity and body image perception on motor skill development.

### 4.1. Reference Values for the Development of Gross Motor Skills in Children

Beyond the significance of establishing normative values to better assess regional children’s motor development, this study has highlighted specific characteristics associated with age and gender. Overall, our findings reveal a period of rapid growth in GMSs between 6 and 9 years old, with subsequent development occurring at a slower rate. This observation is particularly interesting as it underscores the importance of fostering the development of GMSs from an early age when the body is especially receptive to their acquisition. While the prepubescent period is generally considered optimal for GMS acquisition [1,2], our findings suggest that early childhood (before 10 years old) represents a particularly advantageous window for implementing interventions to enhance GMS development.

At the same age, anthropometric measurements between both sexes are similar, although girls tend to be slightly taller and heavier. This is consistent with the well-established phenomenon of earlier physical maturation in girls compared to boys during this developmental stage [90]. Despite similar physical profiles, significant disparities emerge in motor skill development, indicating a weak association between anthropometric measurements and motor skill capacities between boys and girls of this age range. Notably, sex differences vary depending on the specific motor task. For segmental speed tasks, such as one-hand and two-foot tapping, no significant performance differences have been observed between boys and girls. This suggests that isolating segmental movements, a relatively simple task, does not result in significant sex differences, at least prior to puberty. However, it is observed that, in tests requiring the displacement of body mass, boys generally benefit from an advantage. This phenomenon is frequently noted in adolescence and is primarily attributed to a significant increase in muscle mass, which itself results from a substantial rise in sex-related hormones, particularly in testosterone levels [90]. Nevertheless, this explanation appears to be of limited relevance for prepubescent boys. The most plausible explanation lies in the ability to potentiate the contraction force of skeletal muscles [91,92]. Indeed, in boys before puberty, this ability can be attributed to a combination of more effective neuromuscular activation and better muscle coordination.

Conversely, girls outperformed boys in balance tests and movements requiring more complex coordination involving multiple body segments, including contralateral interlimb coordination [93,94,95]. Our results highlighted a significant gender disparity in the development of complex motor skills during childhood. They highlight subtle differences in the acquisition and execution of these skills between boys and girls, providing valuable insights into gender-specific patterns of neuromotor development.

In tests of simple reaction time, ball dribbling, and target ball toss, boys perform better than girls. These results are consistent with other studies reporting similar results [96,97,98]. The reasons for these disparities are unclear. Although some neurobiological characteristics may partly explain these differences, the most plausible causes seem to be sociocultural in nature. Mainly in North America, sports such as baseball and basketball are very popular among boys, even at a very young age. However, the participation rate of girls in this type of activity is rather low. The same goes for video games [99,100], which could partly explain the superiority of boys in tasks involving reaction speed. This phenomenon underscores the complex interplay between biological predispositions and environmental factors in shaping motor skills and cognitive abilities. It highlights the need for a nuanced understanding of gender-based performance differences, emphasizing the significant role that cultural norms, societal expectations, and early exposure to specific activities play in skill development during childhood.

Finally, the results of the 20 m shuttle run test confirm the superior performance of boys, a well-documented phenomenon. This disparity was already observed in Québec in the 1980s [81], at a time when the cardiorespiratory fitness of children—both boys and girls—was significantly higher than it is today [101]. This persistent difference can likely be attributed, in large part, to the generally higher levels of physical activity among boys. Furthermore, as our findings confirm, girls tend to perform less effectively in activities requiring the movement of body mass, a phenomenon that appears to be amplified in prolonged events demanding aerobic capacity.

### 4.2. Impact of Gross Motor Skills Development and Cardiorespiratory Fitness on Body Image Dissatisfaction

Overall, children who have a positive perception of their body image tend to perform better in most motor skills tests compared to those who experience body dissatisfaction. Boys who are dissatisfied with their body image are significantly more affected than girls with similar dissatisfaction, particularly in terms of VO_2_peak values and functional performance, as measured by the number of 1 min stages completed. These findings align with previous studies, which have demonstrated that obese children exhibit fewer motor skills competence and greater body dissatisfaction [71,72]. This trend is especially pronounced when obese boys are compared to their non-obese counterparts [72]. However, our results are not limited to obese children. They encompass children of varying body sizes. As such, these findings represent a meaningful advancement in understanding the relationship between GMSs and body dissatisfaction.

To explain our findings, it is useful to refer to studies that have examined sports dropout, perceived competence, and body dissatisfaction. Research has shown that discontinuing sports during childhood can be partially explained by a child’s perception of their own competence, even when this is not objectively measured [102]. Between the ages of 5 and 11, children compare themselves in terms of physical performance and ability, with this tendency becoming particularly pronounced between ages 9 and 11 [75,77,102,103,104]. A negative self-assessment of their abilities often leads children to reduce or abandon sports participation. By withdrawing from physical activities, they have fewer opportunities to develop their skills and achieve strong performances in physical tests [77], such as those conducted in this study. Engaging in physical activity plays a crucial role in enhancing body satisfaction. In this regard, the limited research available suggests that body dissatisfaction, much like obesity, is a key factor influencing GMSs, particularly in boys [77,105]. To explain this sex difference, the gender stereotype hypothesis has been put forward [77]. From an early age, boys are expected to participate in sports, while girls are socially encouraged not only to engage in sports but also to take part in other activities that promote motor skill development, such as the arts or role-playing. As a result, boys who participate less in sports, partly due to a negative self-assessment of their physical competence, may be more affected in terms of motor skills, as they have fewer opportunities to develop their motor skills.

Finally, it is important to highlight the concerning proportion of children who are dissatisfied with their bodies: 56.8% of boys and 57.3% of girls. Another significant aspect of these findings is the variation in body image satisfaction across different age groups. This satisfaction tends to increase with age, rising from 39.4% to 45.3% between ages 6–8 and 9–12 in boys, with a similar trend observed in girls. Body dissatisfaction affects both children with a typical BMI and those with obesity. Among boys with a typical BMI, 40% of 6–8-year-olds report being dissatisfied with their body image, a figure that increases to 50.8% between ages 9 and 12. For the analyses, a sampling threshold was set at 9 years, based on the child development literature, which highlights developmental differences between early and late childhood. Indeed, self-awareness strengthens with age and cognitive development [106,107].

Our results align with existing research, but the extent of the phenomenon from such a young age (6–8 years) is alarming. They underscore the urgency of early intervention to address this issue, given its numerous harmful consequences [33,44,45,49,50,51,52,53,54,108].

### 4.3. Relationship Between Obesity Status and Gross Motor Skills Competence

Proper acquisition of GMSs is essential for the optimal development of children, particularly before puberty [1,2]. A delay in motor development can have significant consequences, not only in the short term but also extending into adulthood, thereby limiting participation in various activities of daily life, including within the workplace [3,4]. While the relationship between obesity and most aspects of physical fitness is well established, its impact on the development of GMSs remains less clearly understood.

Overall, the present study demonstrates that overweight and/or obesity impact the performance of several components of motor skill development in children, an observation corroborated by numerous studies [14,16,17]. However, our results reveal a considerable disparity in outcomes depending on the specific factor measured. Indeed, in both boys and girls, certain aspects of motor development appear to be minimally or not at all affected by obesity status. Specifically, motor determinants such as segmental speed (arms and feet), simple reaction time, target ball toss, hand–foot coordination, and ball dribbling exhibit neither statistically significant differences (*p* > 0.05) nor notable clinical significance, as evidenced by effect sizes that are generally below f < 0.10.

The primary commonality among these tests is that they require few movements involving significant displacement of a large portion of body mass. In girls, this factor appears particularly important, as all tests involving rapid or prolonged displacement of body mass are affected. The only exception is the slalom run, which is also not impacted in boys. We believe this result can be attributed to the particular difficulty of this test, which led the children to focus primarily on executing the course correctly rather than on speed. As a result, this considerably slowed down all participants, limiting the ability of this test to effectively discriminate based on obesity status. Therefore, it would be more prudent in the future to choose a simpler procedure better suited to individuals in this age group.

While static balance does not require significant shifts in body mass, children who are overweight or obese tend to perform worse on this type of test. Excessive weight gain from an early age, driven by the disproportionate development of fat mass, often results in morphological changes and a decline in postural control, progressively impairing balance ability [20,21,22]. This excess weight can shift the center of gravity, making it more challenging to maintain stability. Additionally, it can lead to structural changes, particularly in the feet, further compromising balance control. These changes may include a collapsed arch, uneven plantar pressure distribution, and increased foot instability. Consequently, the body must compensate through additional postural adjustments, which heighten muscle demand and accelerate fatigue. Over time, these factors can diminish the ability to respond effectively to balance disturbances, making even simple tasks, such as standing on one leg or maintaining a stable posture, significantly more difficult. As a result, overweight and obese children face an increased risk of developing postural control disorders, which can negatively impact their GMSs and reduce their participation in physical activity.

Despite the fact that overweight and obesity undeniably impact children’s motor skills performance, other psychosocial factors may also play a significant role. Indeed, our research has shown that, even among children with a healthy BMI, differences in motor skills emerge depending on their level of body image satisfaction. Our findings suggest that the anthropometric profile alone does not fully account for body image dissatisfaction, emphasizing the need to also consider psychological, cultural, and social dimensions [72,109,110].

Although it is well established that overweight and obese children experience impaired motor performance across various motor domains, the findings of this study highlight that overweight children are significantly less affected than their obese counterparts. Therefore, early intervention for children showing signs of overweight could help slow the decline in their motor skill development. This intervention is crucial, as it is well known that children with low levels of motor development tend to adopt and maintain a sedentary lifestyle [1,12,13,14]. This not only increases their risk of retaining motor sequelae that impact their quality of life but also exposes them to the premature development of cardiometabolic diseases. Timely and targeted action is thus essential to break this vicious cycle and promote their long-term well-being.

#### Strengths and Limits

The present study has several strengths that deserve to be highlighted. Firstly, the large sample size (N = 3144) ensures strong regional representativeness of Canadian children living in Québec. The anthropometric measurements and GMS tests used in this study are well-established and reliable assessment tools. Secondly, the exploration of the relationship between body image dissatisfaction and motor skill development is a novel contribution to research on the Canadian population. Additionally, the inclusion of an independent silhouette assessment enhances the validity of our findings. Furthermore, to our knowledge, this is one of the first Canadian studies to investigate, in greater depth, the associations between GMS development and obesity within a large sample of Canadian children. However, this study also has certain limitations. The cross-sectional design does not allow for the establishment of causal relationships. Although the sample is representative of Québec children, generalization to other Canadian provinces must be approached with caution. Moreover, some tests, such as the slalom race, proved too challenging, especially for the youngest participants. This excessive difficulty limited the ability to accurately assess the impact of this factor across different obesity statuses.

## 5. Conclusions

As evidenced by this study, motor development appears to progress along distinct trajectories in girls and boys. Therefore, the establishment of sex- and age-specific norms is crucial for the accurate assessment of motor function in children. This research highlights gender-based differences, with boys demonstrating superior performance in activities involving body mass displacement, while girls excel in balance tests and movements requiring complex, multi-segment coordination. A positive body image correlates with better performance on motor skills tests. Conversely, boys dissatisfied with their physique are more likely to discontinue sports participation, hindering their motor development. While childhood obesity is generally associated with impaired motor skills, some abilities appear minimally affected. Early intervention for overweight children offers a promising avenue to mitigate declines in motor proficiency and promote an active lifestyle, thereby preventing the establishment of sedentary habits.

## Figures and Tables

**Table 1:** Comparison between boys and girls on anthropometric profile, gross motor skill performance, and cardiorespiratory fitness in children aged 6 to 12 years old.

Variables	Boys	n	Girls	n	*p* Values	Cohen’s d
Age (years)	9.5 ± 1.7	1535	9.4 ± 1.8	1609	0.852	0.06
BM (kg)	33.0 ± 10.3	1280	33.8 ± 11.2	1302	0.061	0.07
BH (cm)	137.5 ± 11.6	1279	138.3 ± 12.8	1302	0.084	0.07
BMI (kg/m^2^)	17.1 ± 3.3	1278	17.3 ± 3.4	1302	0.322	0.06
One-hand plate tapping (Nb/20 s)	66.1 ± 11.8	1440	66.7 ± 12.8	1515	0.170	0.05
Two-foot tapping (Nb/20 s)	21.7 ± 0.4.9	1426	21.9 ± 4.8	1512	0.445	0.04
5 × 5 m shuttle run (s)	11.1 ± 1.3	1421	11.4 ± 1.3	1524	<0.001	0.23
Circle run (s)	22.1 ± 2.7	1441	22.7 ± 2.5	1538	<0.001	0.24
Sidestep run (s)	11.3 ± 1.8	1440	11.7 ± 1.8	1510	<0.001	0.23
Slalom run (s)	19.7 ± 3.3	1428	20.2 ± 3.0	548	<0.001	0.16
Balance eyes opened (s)	23.1 ± 17.9	1439	27.1 ± 19.9	1497	<0.001	0.21
Balance eyes closed (s)	17.7 ± 15.0	1366	20.0 ± 16.4	1428	<0.001	0.15
Simple reaction time (ms)	282.3 ± 19.9	778	288.2 ± 19.5	809	<0.001	0.30
Target ball toss (pts)	4.6 ± 2.9	1457	3.1 ± 2.3	1537	<0.001	0.55
Hand–foot coordination (s)	14.5 ± 7.4	1359	12.6 ± 6.4	1464	<0.001	0.28
Ball dribble (Nb/20 s)	38.3 ± 8.6	1445	35.6 ± 8.4	1528	<0.001	0.32
VO_2_peak (ml/kg/min)	46.3 ± 4.5	979	44.8 ± 3.8	891	<0.001	0.36
Stages (number)	3.5 ± 1.9	980	2.8 ± 1.5	891	<0.001	0.40

n = Number of participants; scores are presented as mean ± SD = standard deviation; *p* values significant at *p* ≤ 0.05; Cohen’s d = effect size: 0.20 = small effect; 0.50 = moderate effect; 0.80 = large effect; BM = body mass; BH = body height; BMI = body mass index.

**Table 2:** Sex-related comparisons of gross motor skills and cardiorespiratory fitness profiles in children with satisfied or dissatisfied body image.

Variables	Satisfied			Dissatisfied				
	n	Mean ± SD	CI	n	Mean ± SD	CI	*p* Values	Cohen’s d ES
Boys								
Age (years)	481	9.8 ± 1.7	9.6–9.9	634	9.6 ± 1.7	9.5–9.7	0.091	0.03
One-hand tapping (Nb/20 s)	468	67.3 ± 11.9	66.3–68.4	622	65.9 ± 11.4	65.0–66.8	0.042	0.12
Two-foot tapping (Nb/20 s)	465	22.7 ± 4.8	22.3–23.2	614	21.6 ± 4.6	21.2–22.0	<0.001	0.24
5 × 5 m shuttle run (s)	450	11.1 ± 1.2	10.9–11.2	603	11.4 ± 1.3	11.3–11.5	<0.001	0.24
Circle run (s)	468	21.8 ± 2.6	21.6–22.0	613	22.3 ± 2.6	22.1–22.5	0.002	0.19
Sidestep run (s)	462	11.1 ± 1.7	10.9–11.2	615	11.4 ± 1.7	11.2–11.5	0.003	0.18
Slalom run (s)	463	19.3 ± 2.2	19.0–19.6	611	19.8 ± 3.3	19.5–20.0	0.025	0.17
Balance eyes opened (s)	463	24.6 ± 18.4	22.9–26.2	620	21.3 ± 17.6	19.9–22.7	0.003	0.18
Balance eyes closed (s)	449	19.3 ± 15.4	17.9–20.8	599	17.9 ± 15.1	16.7–19.1	0.133	0.09
Simple reaction time (ms)	287	283 ± 20	280–285	427	282 ± 20	280–284	0.766	0.05
Target ball toss (Pts)	475	4.8 ± 3.0	4.5–5.1	621	4.5 ± 2.9	4.3–4.7	0.124	0.10
Hand/foot coordination (s)	443	13.5 ± 6.8	12.8–14.1	596	14.8 ± 7.3	14.2–15.3	0.004	0.18
Ball dribble (Nb/20 s)	460	39.3 ± 8.0	38.6–40.0	619	38.0 ± 8.9	37.3–38.7	0.012	0.15
VO_2_peak (ml/kg/min)	381	46.8 ± 4.5	46.3–47.2	528	46.0 ± 4.7	45.6–46.4	0.013	0.17
Stages (number)	381	3.8 ± 1.9	3.6–4.0	528	3.3 ± 1.9	3.2–3.5	<0.001	0.26
Girls								
Age (years)	486	9.8 ± 1.7	9.7–10.0	657	9.7 ± 1.7	9.5–9.8	0.079	0.06
One-hand tapping (Nb/20 s)	470	68.4 ± 13.1	67.2–69.5	635	67.1 ± 12.4	66.1–68.1	0.102	0.10
Two-foot tapping (Nb/20 s)	466	22.8 ± 4.6	22.4–23.2	634	22.2 ± 4.4	21.9–22.5	0.027	0.13
5 × 5 m shuttle run (s)	472	11.3 ± 1.2	11.2–11.4	628	11.6 ± 1.2	11.5–11.7	<0.001	0.25
Circle run (s)	474	22.4 ± 2.4	22.2–22.6	636	22.6 ± 2.4	22.5–22.8	0.073	0.08
Sidestep run (s)	466	11.3 ± 1.7	11.2–11.5	624	11.7 ± 1.7	11.6–11.8	<0.001	0.24
Slalom run (s)	467	19.7 ± 3.0	19.4–20.0	627	20.1 ± 2.9	19.9–20.4	0.022	0.14
Balance eyes opened (s)	466	29.7 ± 20.3	27.9–31.6	625	25.9 ± 19.8	24.3–27.4	0.002	0.19
Balance eyes closed (s)	450	23.5 ± 16.6	22.0–25.1	606	19.8 ± 16.3	18.5–21.1	<0.001	0.23
Simple reaction time (ms)	305	287 ± 20	285–289	433	289 ± 20	287–291	0.131	0.10
Target ball toss (Pts)	476	3.3 ± 2.4	3.0–3.5	630	3.2 ± 2.4	3.0–3.3	0.505	0.04
Hand/foot coordination (s)	451	11.7 ± 5.8	11.1–12.2	620	12.0 ± 5.8	11.6–12.5	0.362	0.05
Ball dribble (Nb/20 s)	465	36.8 ± 7.7	36.1–37.5	628	35.8 ± 8.4	35.1–36.4	0.036	0.12
VO_2_peak (ml/kg/min)	346	44.9 ± 3.8	44.5–45.4	464	44.6 ± 3.8	44.3–45.0	0.261	0.08
Stages (number)	346	3.0 ± 1.5	2.8–3.1	464	2.8 ± 1.4	2.7–3.0	0.118	0.14

n = Number of participants; scores are presented as mean ± SD = standard deviation; CI = confidence interval; *p* values significant at *p* ≤ 0.05; Cohen’s d = effect size: 0.20 = small effect; 0.50 = moderate effect; 0.80 = large effect; ES = effect size.

**Table 3:** Global prevalence of body image dissatisfaction and its associations with BMI, socioeconomic status, and validation of body image self-assessment tool.

Overall Body Image Dissatisfaction				
All Participants	Boys		Girls	
	n = 1076	%	n = 1084	%
Satisfied	465	43.2	463	42.7
Want to be thinner	341	31.7	511	47.1
Want to be bigger	270	25.1	110	10.2
Body image dissatisfaction based on the desire to be thinner or bigger				
Dissatisfied	n = 611	%	n = 620	%
Want to be thinner	341	44.2	511	82.3
Want to be bigger	270	55.8	110	17.7
Body image dissatisfaction vs. BMI				
Typical BMI	n = 909	%	n = 878	%
Satisfied	427	47.0	424	48.3
Dissatisfied	482	53.0	454	51.7
Overweight	n = 108	%	n = 146	%
Satisfied	27	25.0	30	20.6
Dissatisfied	81	75.0	116	79.4
Obese	n = 59	%	n = 59	%
Satisfied	11	18.6	9	15.3
Dissatisfied	48	81.4	50	84.7
Body image dissatisfaction vs. socioeconomic status				
	Mean	SD	Mean	SD
Favorable	1.51	0.50	1.61	0.49
Unfavorable	1.56	0.50	1.62	0.49
*p* values (Student *t*-test)	0.106		0.748	
Validation of self-assessment of body image				
	r (n = 1119)	Kendall’s Tau-b	r (n = 1145)	Kendall’s Tau-b
Independent rating	0.558	0.474	0.624	0.542

BMI = body mass index; n = number of participants; r = Spearman correlation coefficient; % = percentage; SD = standard deviation; *p* values significant at *p* ≤ 0.05.

**Table 4:** Prevalence of body image dissatisfaction and its associations with BMI, socioeconomic status, and validation of body image self-assessment tool for boys across age groups.

Overall Body Image Dissatisfaction				
Boys	6 to 8 years old		9 to 12 years old	
	n = 411	%	n = 704	%
Satisfied	162	39.4	319	45.3
Want to be thinner	122	29.7	231	32.8
Want to be bigger	127	30.9	154	21.9
Body image dissatisfaction based on the desire to be thinner or bigger				
Dissatisfied	n = 249	%	n = 385	%
Want to be thinner	122	49.0	231	60.0
Want to be bigger	127	51.0	154	40.0
Body image dissatisfaction vs. BMI				
Typical BMI	n = 332	%	n = 577	%
Satisfied	134	40.4	293	50.8
Dissatisfied	198	59.6	284	49.2
Overweight	n = 30	%	n = 78	%
Satisfied	11	36.7	16	20.5
Dissatisfied	19	63.3	62	79.5
Obese	n = 22	%	n = 37	%
Satisfied	7	31.8	4	10.8
Dissatisfied	15	68.2	33	89.2
Body image dissatisfaction vs. socioeconomic status				
	Mean	SD	Mean	SD
Favorable	1.62	0.49	1.58	0.49
Unfavorable	1.60	0.49	1.22	0.50
*p* values (Student T-test)	0.662		0.121	
Validation of self-assessment of body image				
	r (n = 367)	Kendall’s Tau-b	r (n = 619)	Kendall’s Tau-b
Independent rating	0.505	0.420	0.581	0.496

BMI = body mass index; n = number of participants; r = Spearman correlation coefficient; % = percentage; SD = standard deviation; *p* values significant at *p* ≤ 0.05.

**Table 5:** Prevalence of body image dissatisfaction and its associations with BMI, socioeconomic status, and validation of body image self-assessment tool for girls across two age groups.

Overall Body Image Dissatisfaction				
Girls	6 to 8 years old		9 to 12 years old	
	n = 400	%	n = 743	%
Satisfied	152	38.0	334	45.0
Want to be thinner	190	47.5	348	46.8
Want to be bigger	58	14.5	61	8.2
Body image dissatisfaction based on the desire to be thinner or bigger				
Dissatisfied	n = 248	%	n = 409	%
Want to be thinner	190	76.6	348	85.1
Want to be bigger	58	23.4	61	14.9
Body image dissatisfaction vs. BMI				
Typical BMI	n = 294	%	n = 585	%
Satisfied	121	41.1	303	51.8
Dissatisfied	173	58.9	282	48.2
Overweight	n = 47	%	n = 99	%
Satisfied	14	29.8	16	16.2
Dissatisfied	33	70.2	83	83.8
Obese	n = 20	%	n = 39	%
Satisfied	4	20.0	5	12.8
Dissatisfied	16	80.0	34	87.2
Body image dissatisfaction vs. socioeconomic status				
	Mean	SD	Mean	SD
Favorable	1.56	0.50	1.57	0.50
Unfavorable	1.66	0.47	1.54	0.50
*p* values (Student *t*-test)	0.045		0.337	
Validation of self-assessment of body image				
	r (n = 348)	Kendall’s Tau-b	r (n = 643)	Kendall’s Tau-b
Independent rating	0.547	0.477	0.651	0.565

BMI = body mass index; n = number of participants; R = Spearman correlation coefficient; % = percentage; SD = standard deviation; *p* values significant at *p* ≤ 0.05.

**Table 6:** Impact of obesity status on the development of motor skills and cardiorespiratory fitness in boys and girls aged 6–12 years.

	n	Typical	n	Overweight	n	Obese	*p* Values	Effect Size *f*
Boys								
Age (years)	1074	9.6 ± 1.7	124	9.9 ± 1.7	66	9.6 ± 1.8	0.193	0.08
One-hand tapping (Nb/20 s)	1019	66.3 ± 11.5	123	66.7 ± 12.1	62	65.0 ± 12.4	0.911	0.07
Two-foot tapping (Nb/20 s)	1017	22.1 ± 4.8	120	21.9 ± 4.8	61	20.6 ± 4.3	0.074	0.15
5 × 5 m shuttle run (s)	993	11.2 ± 1.3	117	11.4 ± 1.2	62	11.8 ± 1.1	0.001	0.24
Circle run (s)	1015	22.1 ± 2.7	120	22.3 ± 2.4	62	22.6 ± 2.5	0.239	0.09
Sidestep run (s)	1009	11.2 ± 1.8	120	11.4 ± 1.5	61	12.0 ± 1.8	0.002	0.22
Slalom run (s)	999	19.7 ± 3.4	119	19.9 ± 3.1	62	20.5 ± 3.0	0.166	0.12
Balance eyes opened (s)	1012	23.9 ± 17.7	119	18.2 ± 17.7	62	12.9 ± 14.5	<0.001	0.31
Balance eyes closed (s)	977	18.6 ± 15.1	113	17.3 ± 15.7	60	11.2 ± 12.6	0.001	0.24
Simple reaction time (ms)	638	283 ± 20	74	281 ± 21	42	279 ± 20	0.467	0.09
Target ball toss (pts)	1029	4.6 ± 3.0	121	4.7 ± 2.9	60	4.8 ± 2.8	0.719	0.03
Hand–eye coordination (s)	972	14.3 ± 7.2	114	14.1 ± 6.3	56	14.9 ± 7.3	0.827	0.06
Ball dribble (Nb/20 s)	1009	38.3 ± 8.7	123	38.8 ± 8.1	63	38.2 ± 8.1	0.808	0.08
Stages (Nb)	825	3.7 ± 1.9	105	2.7± 1.5	48	1.9 ± 0.9	<0.001	0.61
VO_2_peak (ml/kg/min)	824	46.8 ± 4.4	105	44.2 ± 4.0	48	42.2 ± 3.9	<0.001	0.53
Girls								
Age (years)	1059	9.6 ± 1.7	170	9.9 ± 1.7	64	9.8 ± 1.7	0.066	0.08
One-hand tapping (Nb/20 s)	1015	67.5 ± 12.7	162	66.6 ± 12.2	63	65.4 ± 12.1	0.350	0.08
Two-foot tapping (Nb/20 s)	1028	22.2 ± 4.8	162	22.1 ± 4.3	63	21.2 ± 4.0	0.210	0.11
5 × 5 m shuttle run (s)	1015	11.5 ± 1.2	159	11.6 ± 1.1	57	12.0 ± 1.2	0.001	0.20
Circle run (s)	1024	22.6 ± 2.5	164	22.8 ± 2.2	62	23.7 ± 2.3	0.001	0.23
Sidestep run (s)	1002	11.6 ± 1.7	155	11.7 ± 1.5	62	12.3 ± 1.6	0.002	0.21
Slalom run (s)	1002	20.1 ± 3.1	160	20.2 ± 3.0	60	20.5 ± 2.7	0.552	0.06
Balance eyes opened (s)	998	28.5 ± 19.5	159	23.8 ± 20.3	61	16.7 ± 20.4	<0.001	0.28
Balance eyes closed (s)	960	21.3 ± 16.4	153	17.9 ± 16.2	60	16.2 ± 15.3	0.007	0.15
Simple reaction time (ms)	621	288 ± 20	106	289 ± 20	42	289 ± 17	0.929	0.03
Target ball toss (pts)	1022	3.2 ± 2.4	163	3.3 ± 2.2	63	2.7 ± 2.4	0.323	0.12
Hand–eye coordination (s)	985	12.4 ± 6.2	161	12.1 ± 5.2	61	13.1 ± 6.4	0.552	0.08
Ball dribble (Nb/20 s)	1013	35.9 ± 8.4	161	36.2 ± 7.8	62	35.0 ± 7.0	0.616	0.07
Stages (Nb)	721	3.0 ± 1.5	126	2.3 ± 1.1	44	1.9 ± 0.9	<0.001	0.44
VO_2_peak (ml/kg/min)	721	45.2 ± 3.7	126	43.0 ± 3.5	44	42.5 ± 3.4	<0.001	0.36

Nb/20 s = number of repetitions in 20 s; pts = number of points (maximum = 20); n = number of participants; effect size f: 0.10 = small, 0.25 = moderate, 0.40 = large; *p* values significant at *p* ≤ 0.05.

**Table 7:** Comparisons of gross motor skills and cardiorespiratory fitness profiles in children with typical BMI, stratified by sex and body image satisfaction.

Variables	Satisfied			Dissatisfied				
	n	Mean ± SD	CI	n	Mean ± SD	CI	*p* Values	Cohen’s d ES
Boys								
One-hand tapping (Nb/20 s)	415	67.9 ± 11.5	66.8–69.0	473	65.6 ± 11.3	64.6–66.6	0.003	0.20
Two-foot tapping (Nb/20 s)	416	23.0 ± 4.7	22.5–23.4	468	21.7 ± 4.7	21.3–22.1	<0.001	0.28
5 × 5 m shuttle run (s)	403	11.0 ± 1.2	10.9–11.1	461	11.3 ± 1.3	11.3–11.5	<0.001	0.24
Circle run (s)	418	21.8 ± 2.7	21.5–22.0	464	22.3 ± 2.7	22.1–22.6	0.003	0.19
Sidestep run (s)	412	11.0 ± 1.7	10.8–11.3	469	11.3 ± 1.7	11.1–11.4	0.010	0.18
Slalom run (s)	413	19.2 ± 3.3	18.9–19.6	464	19.7 ± 3.3	19.4–20.0	0.037	0.15
Balance eyes opened (s)	414	25.5 ± 18.4	23.7–27.3	473	23.0 ± 17.6	21.4–24.6	0.037	0.14
Balance eyes closed (s)	401	20.1 ± 15.6	18.6–21.6	458	18.6 ± 15.0	17.2–20.0	0.145	0.10
Simple reaction time (ms)	261	283 ± 20	280–285	328	282 ± 20	280–285	0.911	0.01
Target ball toss (Pts)	423	4.9 ± 3.0	4.6–5.2	475	4.5 ± 2.9	4.2–4.7	0.046	0.14
Hand/foot coordination (s)	396	13.1 ± 6.5	12.2–13.8	456	14.8 ± 7.5	14.1–15.5	0.001	0.24
Ball dribble (Nb/20 s)	410	39.7 ± 7.7	39.0–40.5	471	38.0 ± 9.0	37.1–38.8	0.002	0.20
VO_2_peak (ml/kg/min)	351	46.9 ± 4.5	46.5–47.4	417	46.8 ± 4.5	46.3–47.2	0.580	0.02
Stages (number)	351	3.9 ± 1.9	3.7–4.1	417	3.6 ± 1.9	3.4–3.8	0.012	0.16
Girls								
One-hand tapping (Nb/20 s)	411	69.0 ± 12.7	67.8–70.3	439	67.7 ± 12.2	66.5–68.8	0.108	0.10
Two-foot tapping (Nb/20 s)	413	23.1 ± 4.5	22.7–23.5	442	22.4 ± 4.4	22.0–22.8	0.027	0.16
5 × 5 m shuttle run (s)	414	11.3 ± 1.2	11.2–11.4	437	11.5 ± 1.2	11.4–11.6	0.029	0.17
Circle run (s)	417	22.3 ± 2.4	22.1–22.6	441	22.5 ± 2.4	22.3–22.7	0.336	0.09
Sidestep run (s)	408	11.3 ± 1.6	11.1–11.4	432	11.5 ± 1.6	11.4–11.7	0.025	0.14
Slalom run (s)	412	19.6 ± 2.9	19.3–19.9	433	20.0 ± 2.9	19.7–20.3	0.042	0.14
Balance eyes opened (s)	410	30.6 ± 19.9	28.7–32.6	433	28.0 ± 19.3	26.2–29.8	0.050	0.13
Balance eyes closed (s)	396	24.3 ± 16.7	22.6–25.9	418	21.0 ± 16.2	19.4–22.5	0.004	0.20
Simple reaction time (ms)	275	286 ± 19	284–289	296	290 ± 20	287–292	0.042	0.21
Target ball toss (Pts)	417	3.3 ± 2.5	3.1–3.6	435	3.2 ± 2.4	3.0–3.4	0.419	0.04
Hand/foot coordination (s)	397	11.3 ± 5.6	10.8–11.9	430	11.9 ± 5.6	11.3–12.4	0.166	0.11
Ball dribble (Nb/20 s)	411	37.1 ± 7.7	36.3–37.8	434	36.0 ± 8.5	35.2–36.8	0.044	0.14
VO_2_peak (ml/kg/min)	315	45.1 ± 3.8	44.7–45.5	335	45.4 ± 3.6	45.0–45.8	0.337	0.08
Stages (number)	315	3.1 ± 1.5	2.9–3.3	335	3.0 ± 1.4	2.9–3.2	0.707	0.07

n = Number of participants; scores are presented as mean ± SD = standard deviation; *p* values significant at *p* ≤ 0.05; Cohen’s d = effect size: 0.20 = small effect; 0.50 = moderate effect; 0.80 = large effect; ES = effect size.

## Data Availability

The original contributions presented in the study are included in the article/Supplementary Materials. Further inquiries can be directed to the corresponding author.

## Supplementary Materials

The following supporting information can be downloaded at: https://www.mdpi.com/article/10.3390/ijerph22030417/s1. Table S1. Comparison of anthropometric profile, gross motor skill performance, and cardiorespiratory fitness between boys and girls across each chronological age from 6 to 12 years.
