# Supplementary material for: The Relationship Between Obesity Status and Body Image Dissatisfaction on Gross Motor Skill Development and Cardiorespiratory Fitness in Children Aged 6–12 Years Old
Source: Int J Environ Res Public Health. 2025 Mar 12;22(3):417. doi: 10.3390/ijerph22030417 (PMC11942010; doi:10.3390/ijerph22030417)
Supplement: Supplementary file 1 [file ijerph-22-00417-s001.zip › ijerph-3509742-supplementary.pdf]

SUPPLEMENTARY MATERIALS

Comparison of anthropometric profile, gross motor skill performance, and cardiorespiratory fitness between boys and girls across each chronological age from 6 to 12 years.

Variables	Boys			Girls				
	N	Mean ± SD	CI	N	Mean ± SD	CI	P values	Cohen' d
6 years								
Body mass (kg)	83	24.0 ± 6.5	22.5 – 25.4	84	22.4 ± 4.3	21.4 – 23.3	0.061	0.29
Body height (cm)	83	121.1 ± 6.2	119.7 – 122.4	84	120.4 ± 5.7	119.2 – 121.6	0.462	0.12
Body mass index (kg/m^2)	83	16.2 ± 3.0	15.5 – 16.8	84	15.4 ± 2.4	14.9 – 15.9	0.056	0.30
One hand tapping (Nb/20s)	104	51.1 ± 7.8	49.6 - 52.6	114	50.6 ± 9.0	48.9 – 52.2	0.657	0.06
Two foot tapping (Nb/20s)	104	15.3 ± 3.3	14.7 – 15.9	119	15.3 ± 3.1	14.8 – 15.9	0.958	0.00
5X5m shuttle run (s)	101	12.5 ± 1.3	12.2 – 12.7	114	12.8 ± 1.4	12.6 – 13.1	0.064	0.22
Circle run (s)	103	24.3 ± 2.7	23.8 – 24.9	118	24.5 ± 2.2	24.1 – 24.9	0.531	0.08
Side-step run (s)	105	13.6 ± 1.7	13.3 – 13.9	112	13.8 ± 2.1	13.4 – 14.2	0.427	0.10
Slalom run (s)	105	23.7 ± 4.0	23.0 – 24.5	118	23.4 ± 3.4	22.8 – 24.0	0.477	0.08
Balance eyes opened (s)	106	12.6 ± 8.4	11.0 – 14.2	107	13.7 ± 9.7	11.8 – 15.6	0.387	0.12
Balance eyes closed (s)	97	6.8 ± 4.0	6.0 – 7.6	104	8.5 ± 6.3	7.2 – 9.7	**0.026**	0.32
Simple reaction time (ms)	47	294 ± 21	288 – 300	41	302 ± 16	297 – 307	**0.042**	0.45
Target ball toss (Pts)	106	2.0 ± 1.7	1.7 – 2.3	117	1.1 ± 1.0	0.9 -1.3	**<0.001**	0.65
Hand/foot coordination (s)	91	26.4 ± 10.7	24.2 – 28.6	101	21.6 ± 8.3	20.0 – 23.3	**0.001**	0.51
Ball dribble (Nb/20s)	103	29.9 ± 9.2	28.1 – 31.8	118	26.4 ± 9.9	24.6 – 28.2	**0.007**	0.37
VO_2peak (ml/kg/min)	56	48.4 ± 2.3	47.8 – 49.1	59	47.7 ± 2.2	47.1 – 48.3	0.093	0.31
Stages (number)	57	2.2 ± 1.2	1.9 – 2.5	59	1.8 ± 0.9	1.5 – 2.0	**0.030**	0.28
7 years								
Body mass (kg)	169	25.6 ± 5.9	24.7 – 26.5	183	24.6 ± 4.8	23.9 – 25.3	0.065	0.18
Body height (cm)	169	125.5 ± 5.9	124.6 – 126.4	183	124.8 ± 5.7	123.9 – 125.6	0.226	0.12
Body mass index (kg/m^2)	169	16.2 ± 3.1	15.7 – 16.6	183	15.7 ± 2.2	15.4 – 16.0	0.076	0.19
One hand tapping (Nb/20s)	195	55.6 ± 8.1	54.5 – 56.8	228	56.3 ± 8.5	55.2 – 57.4	0.445	0.08
Two foot tapping (Nb/20s)	208	18.2 ± 4.2	17.6 – 18.7	233	17.9 ± 3.9	17.4 – 18.4	0.468	0.07
5X5m shuttle run (s)	201	11.9 ± 1.4	11.7 – 12.1	233	12.2 ± 1.2	12.1 – 12.4	**0.021**	0.23
Circle run (s)	209	23.8 ± 2.9	23.4 – 24.2	239	24.4 ± 2.5	24.1 – 24.7	**0.035**	0.22
Side-step run (s)	205	12.4 ± 1.9	12.2 – 12.7	232	12.9 ± 1.6	12.7 – 13.1	**0.010**	0.29
Slalom run (s)	202	21.7 ± 3.5	21.2 – 22.1	236	22.3 ± 3.1	21.9 – 22.7	0.056	0.18
Balance eyes opened (s)	197	13.3 ± 9.9	12.0 – 14.7	222	17.0 ± 12.4	15.4 -18.6	**0.001**	0.33

Balance eyes closed (s)	194	10.8 ± 7.5	9.7 – 11.8	202	12.5 ± 9.2	11.2 – 13.8	**0.043**	0.20
Simple reaction time (ms)	113	296 ± 15	293 - 299	106	301 ± 16	298 – 304	**0.024**	0.31
Target ball toss (Pts)	201	2.8 ± 2.0	2.5 – 3.0	235	1.7 ± 1.6	1.5 – 1.9	**<0.001**	0.61
Hand/foot coordination (s)	188	18.7 ± 7.1	17.7 – 19.7	219	17.7 ± 7.5	16.7 – 18.7	0.157	0.14
Ball dribble (Nb/20s)	203	32.5 ± 8.3	31.4 – 33.7	235	29.9 ± 9.0	28.8 – 31.1	**0.002**	0.29
VO_2peak (ml/kg/min)	127	48.0 ± 2.9	47.5 – 48.5	117	47.1 ± 2.6	46.7 – 47.6	**0.017**	0.33
Stages (number)	127	2.5 ± 1.3	2.3 – 2.7	117	2.2 ± 1.1	2.0 – 2.4	**0.030**	0.25

Variables	Boys			Girls				
	N	Mean ± SD	CI	N	Mean ± SD	CI	P values	Cohen' d
8 years								
Body mass (kg)	234	28.5 ± 6.7	27.7 – 29.4	216	28.7 ± 7.4	27.7 – 29.7	0.749	0.03
Body height (cm)	234	131.3 ± 6.5	130.5 – 132.1	216	130.8 ± 6.3	130.0 – 132.1	0.422	0.08
Body mass index (kg/m^2)	234	16.4 ± 2.7	16.1 – 16.7	216	16.7 ± 3.6	16.2 – 17.2	0.342	0.10
Upper limb speed ($nb·20s^{-1}$)	266	62.3 ± 8.4	61.3 - 63.3	262	61.9 ± 8.0	60.9 - 62.8	0.545	0.05
Lower limb speed ($nb·20s^{-1}$)	265	20.2 ± 4.1	19.7 - 20.7	263	20.5 ± 3.5	20.1 - 20.9	0.332	0.08
5m shuttle run (s)	263	11.3 ± 1.1	11.2 - 11.5	262	11.6 ± 1.1	11.5 - 11.8	**0.001**	0.27
Circle run (s)	268	22.5 ± 2.4	22.2 - 22.8	265	22.9 ± 2.2	22.7 - 23.2	**0.030**	0.17
Side-step run (s)	266	11.5 ± 1.5	11.3 - 11.7	265	12.0 ± 1.5	11.8 - 12.2	**<0.001**	0.33
Slalom run (s)	265	20.4 ± 2.9	20.0 - 20.7	261	20.8 ± 2.6	20.5 - 21.1	0.119	0.14
Balance eyes opened (s)	265	21.3 ± 16.3	19.4 - 23.3	264	25.6 ± 19.1	23.3 - 27.9	**0.006**	0.24
Balance eyes closed (s)	244	13.9 ± 10.0	12.7 - 15.2	243	15.5 ± 12.0	14.0 - 17.0	0.121	0.15
Simple reaction time (ms)	136	290 ± 17	287 - 293	135	298 ± 15	295 - 300	**<0.001**	0.49
Target ball toss (Pts)	268	3.7 ± 2.4	3.4 - 4.0	264	2.5 ± 1.9	2.3 - 2.8	**<0.001**	0.55
Hand/foot coordination (s)	253	16.3 ± 7.1	15.4 - 17.2	254	13.3 ± 5.2	12.6 - 13.9	**<0.001**	0.48
Ball dribble ($nb·20s^{-1}$)	268	36.4 ± 8.1	35.4 - 37.3	265	34.6 ± 7.1	33. - 35.5	**0.008**	0.24
VO_2peak (ml/kg/min)	166	47.5 ± 4.0	46.9 – 48.1	142	46.3 ± 3.1	45.8 – 48.1	**0.004**	0.33
Stages (number)	142	3.2 ± 1.7	2.9 – 3.5	142	2.6 ± 1.3	2.4 – 2.8	**0.001**	0.39
9 years								
Body mass (kg)	224	31.2 ± 8.1	30.1 – 32.3	231	31.4 ± 7.2	30.4 – 32.3	0.831	0.03
Body height (cm)	223	136.4 ± 7.2	135.5 – 137.3	231	136.2 ± 7.0	135.2 – 137.1	0.713	0.03
Body mass index (kg/m^2)	223	16.6 ± 3.0	16.2 – 17.0	231	16.8 ± 2.7	16.4 – 17.1	0.623	0.07
Upper limb speed ($nb·20s^{-1}$)	250	65.9 ± 8.8	64.8 – 66.9	274	66.5 ± 9.0	65.5 – 67.6	0.388	0.07
Lower limb speed ($nb·20s^{-1}$)	249	22.3 ± 3.7	21.9 – 22.8	268	22.5 ± 3.3	22.1 – 22.9	0.476	0.06

5m shuttle run (s)	256	11.0 ± 1.3	10.9 – 11.2	277	11.3 ± 1.1	11.2 – 11.5	**0.003**	0.25
Circle run (s)	249	22.1 ± 2.4	21.8 – 22.4	274	22.5 ± 2.3	22.3 – 22.8	**0.040**	0.17
Side-step run (s)	254	11.0 ± 1.4	10.8 – 11.2	271	11.5 ± 1.4	11.3 – 11.6	**<0.001**	0.36
Slalom run (s)	248	19.4 ± 2.8	19.1 – 19.8	272	20.1 ± 2.6	19.8 – 20.4	**0.007**	0.26
Balance eyes opened (s)	254	24.2 ± 17.9	22.0 – 26.4	271	28.4 ± 19.7	26.0 – 30.7	**0.011**	0.22
Balance eyes closed (s)	235	15.4 ± 11.2	14.0 – 16.9	264	19.5 ± 14.3	17.7 – 21.2	**<0.001**	0.32
Simple reaction time (ms)	131	283 ± 17	280 – 285	149	291 ± 17	288 – 294	**<0.001**	0.48
Target ball toss (Pts)	260	4.6 ± 2.8	4.3 – 5.0	275	2.7 ± 1.9	2.4 – 2.9	**<0.001**	0.92
Hand/foot coordination (s)	248	13.6 ± 5.9	12.9 – 14.3	267	12.1 ± 5.0	11.5 – 12.7	**0.002**	0.28
Ball dribble ($nb \cdot 20s^{-1}$)	255	39.5 ± 7.3	38.6 – 40.4	273	36.6 ± 6.7	35.8 – 37.4	**<0.001**	0.42
VO_2peak (ml/kg/min)	174	46.7 ± 2.9	46.0 – 47.3	178	44.6 ± 2.9	44.2 – 45.1	**<0.001**	0.72
Stages (number)	174	3.5 ± 1.8	3.2 – 3.8	178	2.7 ± 1.2	2.5 – 2.9	**<0.001**	0.52

Variables	Boys			Girls				
	N	Mean ± SD	CI	N	Mean ± SD	CI	P values	Cohen' d
10 years								
Body mass (kg)	238	35.5 ± 8.0	34.4 – 36.5	226	36.9 ± 9.0	35.7 – 38.1	0.068	0.17
Body height (cm)	237	141.9 ± 6.7	142.6 – 144.7	226	143.7 ± 8.3	142.6 – 144.7	**0.013**	0.24
Body mass index (kg/m^2)	237	17.5 ± 3.2	17.0 – 17.9	226	17.7 ± 3.4	17.3 – 18.2	0.373	0.06
Upper limb speed ($nb \cdot 20s^{-1}$)	273	70.7 ± 9.2	69.6 – 71.8	248	71.9 ± 10.6	70.6 -0 73.3	0.143	0.12
Lower limb speed ($nb \cdot 20s^{-1}$)	256	23.7 ± 3.7	23.2 – 24.2	247	23.9 ± 3.2	23.5 -24.3	0.617	0.06
5m shuttle run (s)	264	10.8 ± 1.1	10.6 – 10.9	253	11.0 ± 1.0	10.9 – 11.2	**0.002**	0.19
Circle run (s)	261	21.2 ± 2.1	21.0 – 21.5	249	22.1 ± 2.2	21.8 – 22.3	**0.000**	0.47
Sidestep run (s)	270	10.8 ± 1.4	10.6 – 10.9	247	11.0 ± 1.3	10.9 – 11.2	**0.025**	0.15
Slalom run (s)	266	18.8 ± 2.4	18.5 – 19.1	245	19.1 ± 2.1	18.9 – 19.4	0.100	0.13
Balance eyes opened (s)	271	27.7 ± 19.2	25.4 – 30.0	250	34.7 ± 20.9	32.1 – 37.3	**<0.001**	0.35
Balance eyes closed (s)	261	21.9 ± 16.2	19.9 – 23.9	246	26.6 ± 18.8	24.3 – 29.0	**0.003**	0.27
Simple reaction time (ms)	144	277 ± 18	274 – 280	132	283 ± 20	280 – 286	**0.011**	0.31
Target ball toss (Pts)	271	5.3 ± 2.7	5.0 – 5.6	253	4.1 ± 2.2	3.8 – 4.4	**<0.001**	0.49
Hand/foot coordination (s)	255	11.7 ± 4.2	11.2 – 12.2	246	9.9 ± 3.5	9.5 – 10.4	**<0.001**	0.47
Ball dribble ($nb \cdot 20s^{-1}$)	268	40.5 ± 6.7	39.7 – 41.3	254	38.8 ± 6.4	38.0 – 39.6	**0.003**	0.26
VO_2peak (ml/kg/min)	198	45.9 ± 4.9	45.3 – 46.6	164	44.1 ± 3.8	43.5 – 44.7	**<0.001**	0.41
Stages (number)	198	3.9 ± 2.0	3.6 – 4.2	164	3.1 ± 1.6	2.9 – 3.4	**<0.001**	0.44
11 years								

Body mass (kg)	190	40.7 ± 10.1	39.3 – 42.2	234	42.5 ± 9.8	41.2 – 43.8	0.067	0.18
Body height (cm)	191	148.2 ± 7.3	147.2 – 149.2	234	150.9 ± 8.0	149.9 – 151.9	**<0.001**	0.35
Body mass index (kg/m^2)	190	18.4 ± 3.6	17.9 – 18.9	234	18.5 ± 3.5	18.1 – 19.0	0.684	0.03
Upper limb speed (nb·20s^{-1})	213	75.0 ± 9.7	73.7 – 76.3	264	77.0 ± 10.5	75.7 – 78.3	**0.032**	0.20
Lower limb speed (nb·20s^{-1})	212	24.7 ± 3.8	24.2 – 25.3	260	25.2 ± 3.6	24.7 – 25.6	0.214	0.14
5m shuttle run (s)	209	10.5 ± 1.1	10.4 – 10.7	262	10.7 ± 1.0	10.6 – 10.9	**0.040**	0.19
Circle run (s)	211	21.1 ± 2.2	20.8 – 21.4	262	21.6 ± 2.0	21.3 – 21.8	**0.010**	0.24
Sidestep run (s)	208	10.3 ± 1.4	10.2 – 10.5	264	10.6 ± 1.3	10.5 – 10.8	**0.020**	0.22
Slalom run (s)	209	18.1 ± 2.2	17.8 – 18.4	261	18.5 ± 1.9	18.3 – 18.7	**0.048**	0.20
Balance eyes opened (s)	210	29.4 ± 19.8	26.7 – 32.1	262	31.0 ± 21.2	28.4 – 33.6	0.406	0.08
Balance eyes closed (s)	208	22.6 ± 17.3	20.3 – 25.0	254	24.6 ± 18.3	22.3 – 26.8	0.249	0.11
Simple reaction time (ms)	120	271 ± 20	267 – 274	163	278 ± 17	275 – 280	**0.001**	0.39
Target ball toss (Pts)	213	6.2 ± 3.0	5.8 – 6.6	264	4.3 ± 2.3	4.0 – 4.6	**<0.001**	0.72
Hand/foot coordination (s)	204	10.5 ± 3.3	10.1 – 11.0	259	9.0 ± 2.7	8.6 – 9.3	**<0.001**	0.50
Ball dribble (nb·20s^{-1})	214	42.2 ± 6.8	41.2 – 43.1	267	39.3 ± 6.1	38.6 – 40.1	**<0.001**	0.45
VO_2peak (ml/kg/min)	154	44.0 ± 4.7	43.3 – 44.7	148	42.6 ± 3.5	42.1 – 43.2	**0.004**	0.34
Stages (number)	154	3.8 ± 1.9	3.5 – 4.1	148	3.3 ± 1.7	3.1 – 3.5	**0.003**	0.28
Variables	Boys			Girls				
	N	Mean ± SD	CI	N	Mean ± SD	CI	P values	Cohen’ d
12 years								
Body mass (kg)	129	44.2 ± 11.3	42.3 – 46.2	118	47.2 ± 11.8	45.1 – 49.3	**0.045**	0.26
Body height (cm)	129	152.6 ± 8.4	151.1 – 154.0	118	153.4 ± 7.0	153.1 – 155.6	0.070	0.10
Body mass index (kg/m^2)	129	18.8 ± 3.7	18.2 – 19.5	118	19.7 ± 4.1	19.0 – 20.4	0.081	0.23
Upper limb speed (nb·20s^{-1})	139	76.7 ± 9.5	75.1 – 78.3	125	78.6 ± 10.6	76.7 – 80.5	0.129	0.19
Lower limb speed (nb·20s^{-1})	132	25.9 ± 4.0	25.2 – 26.6	122	26.5 ± 3.9	25.8 – 27.2	0.285	0.15
5m shuttle run (s)	127	10.3 ± 0.88	10.2 – 10.5	123	10.8 ± 0.98	10.6 – 11.0	**<0.001**	0.54
Circle run (s)	141	20.6 ± 2.1	20.3 – 20.9	130	21.4 ± 2.1	21.1 – 21.8	**0.001**	0.38
Side-step run (s)	132	10.0 ± 1.2	9.8 – 10.2	119	10.4 ± 1.1	10.2 – 10.6	**0.006**	0.35
Slalom run (s)	133	17.1 ± 2.2	16.7 – 17.4	122	18.0 ± 2.3	17.6 – 18.4	**0.001**	0.40
Balance eyes opened (s)	136	27.6 ± 19.8	24.2 – 30.9	121	34.1 ± 21.7	30.2 – 38.0	**0.013**	0.31
Balance eyes closed (s)	127	31.6 ± 20.4	28.1 – 35.2	115	29.8 ± 19.8	26.1 – 33.4	0.474	0.09
Simple reaction time (ms)	86	269 ± 18	266 – 273	83	274 ± 18	270 – 278	0.082	0.28
Target ball toss (Pts)	138	6.6 ± 2.9	6.1 – 7.1	129	5.2 ± 2.6	4.7 – 5.7	**<0.001**	0.51
Hand/foot coordination (s)	120	10.0 ± 3.3	9.4 – 10.6	118	8.5 ± 2.4	8.1 – 8.9	**<0.001**	0.52

Ball dribble (nb·20s^{-1})	134	44.2 ± 7.6	42.9 – 45.5	116	40.9 ± 6.5	39.8 – 42.1	**<0.001**	0.46
VO_2peak (ml/kg/min)	104	44.6 ± 5.0	43.6 – 45.6	83	42.2 ± 4.9	41.1 – 43.3	**0.001**	0.65
Stages (number)	104	4.6 ± 2.0	4.3 – 5.0	83	3.7 ± 1.8	3.3 – 4.1	**0.001**	0.47
